# Functional genomics of corrinoid starvation in the organohalide-respiring bacterium *Dehalobacter restrictus* strain PER-K23

**DOI:** 10.3389/fmicb.2014.00751

**Published:** 2015-01-06

**Authors:** Aamani Rupakula, Yue Lu, Thomas Kruse, Sjef Boeren, Christof Holliger, Hauke Smidt, Julien Maillard

**Affiliations:** ^1^Laboratory for Environmental Biotechnology, ENAC-IIE-LBE, Ecole Polytechnique Fédérale de LausanneLausanne, Switzerland; ^2^Laboratory of Microbiology, Agrotechnology and Food Sciences, Wageningen UniversityWageningen, Netherlands; ^3^Laboratory of Biochemistry, Agrotechnology and Food Sciences, Wageningen UniversityWageningen, Netherlands

**Keywords:** corrinoid biosynthesis, organohalide respiration, *Dehalobacter*, cobalamin riboswitches, functional genomics

## Abstract

*De novo* corrinoid biosynthesis represents one of the most complicated metabolic pathways in nature. Organohalide-respiring bacteria (OHRB) have developed different strategies to deal with their need of corrinoid, as it is an essential cofactor of reductive dehalogenases, the key enzymes in OHR metabolism. In contrast to *Dehalococcoides mccartyi*, the genome of *Dehalobacter restrictus* strain PER-K23 contains a complete set of corrinoid biosynthetic genes, of which *cbiH* appears to be truncated and therefore non-functional, possibly explaining the corrinoid auxotrophy of this obligate OHRB. Comparative genomics within *Dehalobacter* spp. revealed that one (operon-2) of the five distinct corrinoid biosynthesis associated operons present in the genome of *D. restrictus* appeared to be present only in that particular strain, which encodes multiple members of corrinoid transporters and salvaging enzymes. Operon-2 was highly up-regulated upon corrinoid starvation both at the transcriptional (346-fold) and proteomic level (46-fold on average), in line with the presence of an upstream cobalamin riboswitch. Together, these data highlight the importance of this operon in corrinoid homeostasis in *D. restrictus* and the augmented salvaging strategy this bacterium adopted to cope with the need for this essential cofactor.

## Introduction

Corrinoids are essential cofactors for a wide variety of enzymes that facilitate reactions including rearrangements, methyl group transfers, and reductive dehalogenation (Banerjee and Ragsdale, 2003). A recent bioinformatic study has revealed that while 76% of 540 sequenced bacterial genomes contain corrinoid-dependent enzymes, only 39% of these genomes encode the complete corrinoid biosynthesis pathway, suggesting that the salvage of corrinoids from the environment is an important process for many bacteria (Zhang et al., 2009). Both aerobic and anaerobic corrinoid biosynthesis pathways have been described showing few but significant differences, notably in tetrapyrrole ring contraction and the step at which cobalt is inserted into the ring (Scott, 2003; Moore and Warren, 2012). This pathway is complex and consists of approximately 30 reactions (see Moore and Warren, 2012 for a recent review).

Organohalide respiration (OHR) is an anaerobic bacterial respiration process of environmental interest, as many anthropogenic halogenated organic compounds can be used as terminal electron acceptors by organohalide-respiring bacteria (OHRB) (Leys et al., 2013). OHRB are capable to remove the halogens and therefore contribute to bioremediation of environments polluted with these compounds (Smidt and de Vos, 2004). The key enzyme in OHR is the reductive dehalogenase (RDase) (Hug et al., 2013), which strictly depends on corrinoid cofactors for the dehalogenation reaction. Although the reaction mechanism has not yet been fully understood, RDases represent a particular family of corrinoid enzymes as they catalyze electron transfer rather than methyl transfer. Moreover, the absence of a corrinoid binding motif in RDase sequences reflects the base-off/his-off conformation of the corrinoid in the enzyme (Schumacher et al., 1997; van de Pas et al., 1999). In recent years, corrinoid biosynthesis and salvaging in OHRB regained substantial interest in the scientific community as exemplified by the following studies: an unusual corrinoid cofactor (norpseudo-B_12_) has been identified in the tetrachloroethene (PCE) RDase of *Sulfurospirillum multivorans* (Kräutler et al., 2003); the lack of exogenous corrinoid had an effect on the RDase activity of *Desulfitobacterium hafniense* when cultivated with an alternative electron acceptor (Reinhold et al., 2012); many essential corrinoid biosynthetic genes have been found on a plasmid in *Geobacter lovleyi* (Wagner et al., 2012); the involvement of the bacterial community accompanying members of *Dehalococcoides mccartyi* for corrinoid supply has been highlighted (Hug et al., 2012; Yan et al., 2012, 2013; Men et al., 2014b); modifying the lower ligand of the corrinoid had a severe effect on the activity of the PCE RDase of *S. multivorans* (Keller et al., 2013).

Contrasting situations have been observed regarding the ability of OHRB to produce corrinoid cofactors *de novo*. Both genome analysis and physiological studies have shown that the obligate OHRB *D. mccartyi* is strictly dependent on exogenous corrinoid supply and that 5,6-dimethylbenzimidazole can serve as nucleotide loop in corrinoid cofactors (Yi et al., 2012; Löffler et al., 2013; Yan et al., 2013; Men et al., 2014a,b). On the contrary, the facultative OHRB *S. multivorans* strain K and *D. hafniense* strains encode the full corrinoid biosynthetic pathway in their genome and have been shown to grow without any supply of corrinoid in the medium (Nonaka et al., 2006; Kim et al., 2012; Choudhary et al., 2013; Goris et al., 2014).

**Figure 1:**
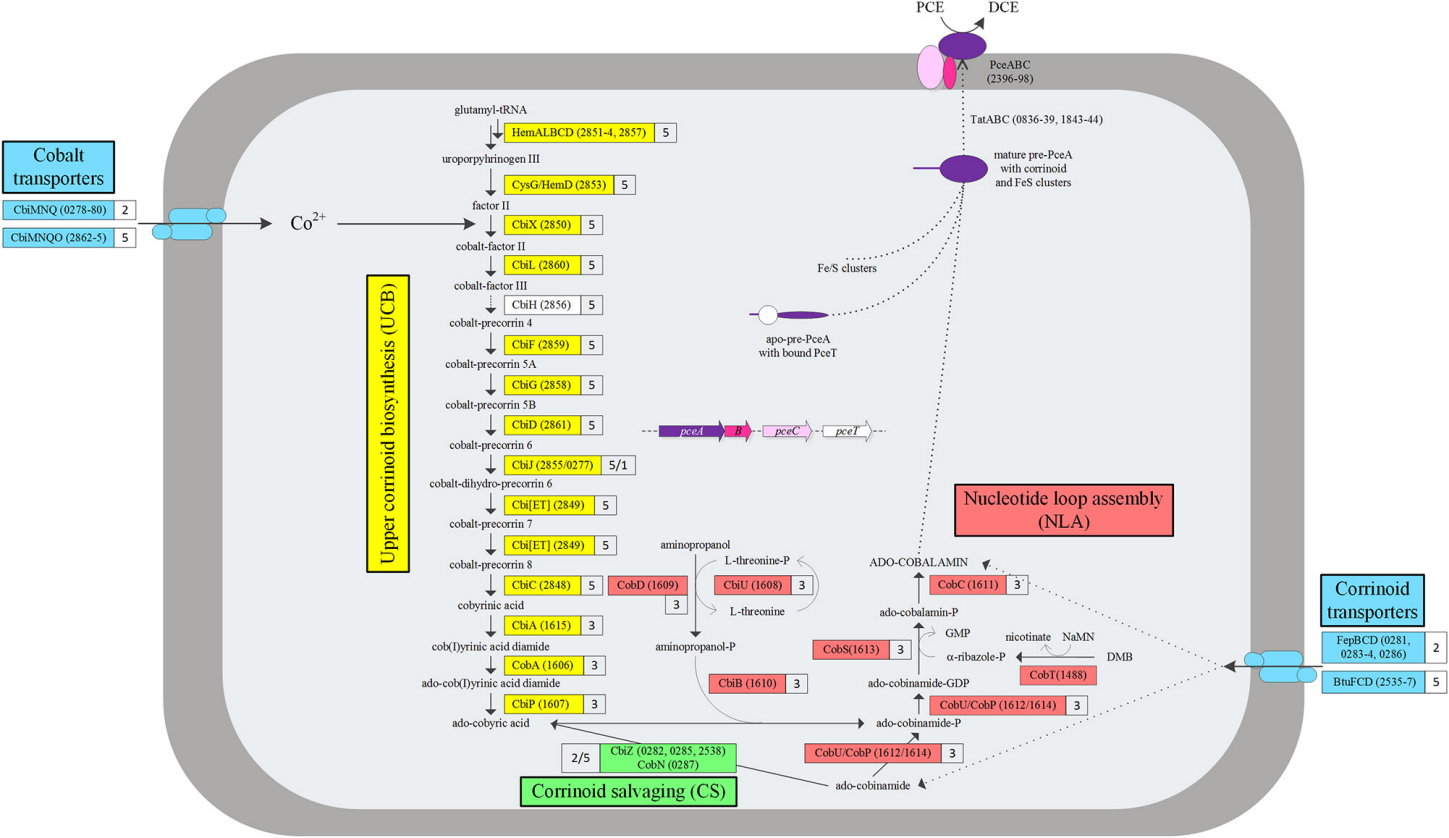
**Predicted corrinoid biosynthesis pathway of *D. restrictus***. Based on the genome annotation, a map of the corrinoid biosynthesis was predicted including four pathways: the upper corrinoid biosynthesis (UCB, in yellow), the nucleotide loop assembly (NLA, in red), cobalt and corrinoid transporters (in blue) and corrinoid salvage (in green). The PCE reductive dehalogenase (PceA and associated proteins PceB and PceC) is also depicted. The enzymes catalyzing each reaction are given as color boxes indicating the protein name (when available) and corresponding gene loci (with the Dehre_# prefix removed). CbiH in *D. restrictus* is likely to be non-functional as the corresponding gene is truncated (white box). The operon number to which every locus belongs is indicated besides each protein. Note: the details of CbiH catalyzed reaction can be found in (Moore and Warren, 2012).

*Dehalobacter restrictus* strain PER-K23 is an obligate OHRB only able to grow by dechlorinating tetra- and trichloroethene (PCE and TCE, respectively). It was first isolated from Rhine river sediment and since then always cultivated in the presence of exogenous vitamin B_12_ (cyanocobalamin) (Holliger et al., 1998). The PCE RDase (PceA) of *D. restrictus* has been extensively studied and revealed a 60-kDa enzyme containing a corrinoid cofactor and two 4Fe-4S clusters with estimated redox potential of −350 mV (Co^1+/2+^) and −480 mV (4Fe-4S^2+/1+^), respectively, and a specific dechlorination activity of 250 nkat/mg (Schumacher et al., 1997; Maillard et al., 2003). Spectrophotometric analysis of the corrinoid extracted from *D. restrictus* PceA with cyanide has shown a spectrum resembling the one of cyanocobalamin (Schumacher et al., 1997), although this method does not allow identifying corrinoid unambiguously. Analysis of the newly published genome of *D. restrictus* revealed the presence of a complete set of corrinoid biosynthetic genes where one gene, *cbiH*, is truncated due to a 101-bp deletion, likely responsible for the corrinoid auxotrophy of *D. restrictus* (Kruse et al., 2013; Rupakula et al., 2013) (Figure 1).

The present study aims to explore in detail the effect of corrinoid starvation on *D. restrictus* with a combination of comparative genomics, as well as transcription and proteome analysis.

## Materials and methods

### Bacteria, plasmids, and growth conditions

*D. restrictus* strain PER-K23 (DSM 9455) was cultivated as described earlier (Holliger et al., 1998; Maillard et al., 2003; Rupakula et al., 2013). Anaerobic serum flasks of 500 mL were supplemented with hydrogen as electron donor, inoculated with 2% (v/v) inoculum, and finally 1% (v/v) of 2 M PCE dissolved in hexadecane was added as electron acceptor. Batch cultures of *D. restrictus* were cultivated in 300 mL medium at 30°C under gentle agitation (100 rpm), and chloride release was used as an indicator of growth. Chloride concentration was measured with a Chlor-o-counter (Flohr Instrument, Nieuwegein, Netherlands) as described earlier (Maillard et al., 2011). Cultures for proteomic analysis were prepared in triplicate with high, mid, and low concentration of cyanocobalamin corresponding to 250, 50, and 10 μg/L, respectively.

*Escherichia coli* DH5α was cultivated in liquid or solid LB medium containing 100 μg/L ampicillin after transformation with derivatives of the pGEM-T Easy vector (Promega, Duebendorf, Switzerland).

### Sequence retrieval and genome analysis

All sequences mentioned in this study were taken from the recently published genome of *D. restrictus* strain PER-K23 (Kruse et al., 2013) and from other *Dehalobacter* spp. genomes including *Dehalobacter* sp. E1 (Maphosa et al., 2012), *Dehalobacter* sp. DCA and sp. CF (Tang et al., 2012), *Dehalobacter* sp. FTH1 (RefSeq PRJNA199134, JGI genome project), *Dehalobacter* sp. UNSWDHB (Deshpande et al., 2013). The original annotation of *D. restrictus* gene loci obtained in collaboration with the Joint Genome Institute (JGI project #402027) was used here as the present study is the follow-up study of two previous reports where the JGI annotation was used (Kruse et al., 2013; Rupakula et al., 2013). Another version of *D. restrictus* genome was recently annotated by the automatic pipeline of the NCBI database and is available under accession number CP007033. Corresponding loci from both databases are given for the selected corrinoid proteome in Table S1.

The annotation of selected genes was verified using a manual search with BLAST (Altschul et al., 1990). Protein sequences were aligned using ClustalX v.2.0 (Larkin et al., 2007). Sequence maximum likelihood tree analysis was done with MEGA5 (Tamura et al., 2011). Cobalamin riboswitches (Cbl-RS) were identified using Rfam (Burge et al., 2013) and initially aligned using ClustalX and then corrected manually as described earlier (Choudhary et al., 2013). Comparative genome analysis was performed using the Artemis Comparison Tool (Carver et al., 2005).

### Transcription analysis

RNA was extracted using the TRIzol method according to (Prat et al., 2012) with the following modification. The DNaseI treatment was stopped by adding the DNase stop solution and incubating for 10 min at 65°C. RNA concentration was estimated using the Nanodrop ND-1000 spectrophotometer (Thermo Scientific, Ecublens, Switzerland). Reverse transcription was performed as described in Rupakula et al. (2013).

Primers targeting each gene present immediately downstream of the five Cbl-RS of *D. restrictus* were designed. PCRs and cloning using the pGEM-T Easy vector, clone selection, sequencing and quantitative PCR were performed as described earlier (Rupakula et al., 2013). Primer sequences, amplicon sizes, and plasmids are given in Table S2.

### Protein extraction and SDS-PAGE

Cells were harvested by 10 min centrifugation at 12000× *g*, washed twice with 25 mL 20 mM Tris-HCl (pH 7.5), and then flash-frozen in liquid nitrogen. All biomass samples were stored at −80°C until use. Cell pellets were resuspended in 0.5 mL lysis buffer (100 mM Tris/HCl, pH 7.5, 4% sodium dodecyl sulfate, and 0.1 M dithiothreitol) and then transferred to 2-mL protein LoBind tubes (Eppendorf, Hamburg, Germany). Protein extraction was done as described earlier (Rupakula et al., 2013). Protein concentration was determined with the Qubit® protein assay kit (Invitrogen, Eugene, OR, USA) following the manufacturer's instructions. Protein samples were stored at −20°C until use. SDS-PAGE was done following standard procedures (Sambrook et al., 1989). In brief, 15 μg of proteins from each sample were loaded in separate lanes in gels containing 10% SDS. Gels were stained with Coomassie brilliant blue R250 (Merck, Darmstadt, Germany) and scanned using a GS-800 calibrated densitometer (Bio-Rad, Hercules, CA, USA). The Quantity One basic software package was used to quantify the intensity of lanes. Series of gels were prepared and analyzed until less than 5% differences in the intensity between any lanes were achieved.

### Gel digestion and peptides purification

In-gel digestion of proteins and purification of peptides were done following a modified version of the protocol described earlier (Rupakula et al., 2013). Disulphide bridges in proteins were reduced by covering whole gels with reducing solution (10 mM dithiothreitol, pH 7.6, in 50 mM NH_4_HCO_3_), and the gels were incubated at 60°C for 1 h. Alkylation was performed for 1 h by adding 25 mL of iodoacetamide solution (10 mM iodoacetamide in 100 mM Tris-HCl, pH 8.0). Gels were thoroughly rinsed with dd H_2_O water in between steps. Each lane of SDS-PAGE gels was cut into three equally sized slices, and each slice was cut into approximately 1 mm^3^ cubes and transferred to separate 0.5 mL protein LoBind tubes (Eppendorf, Hamburg, Germany). Enzymatic digestion was done by adding 50 μL of trypsin solution (5 ng/μL trypsin in 50 mM NH_4_HCO_3_) to each tube, and by incubating at room temperature overnight with gentle shaking. Extraction of peptides was performed with manual sonication in an ultrasonic water bath for 1 s before the supernatant was transferred to a clean protein LoBind tube. Additional peptides were recovered by adding 25 μL of 2.5% (v/v) trifluoroacetic acid to the gel pieces, which were sonicated for 2 s before the supernatant was combined with the first supernatant obtained. Peptides were purified with a C18 Empore disk as previously described (Rappsilber et al., 2007). Acetonitrile in the samples was removed by using a concentrator vacuum centrifuge. Finally, sample volume was adjusted to 50 μL with 0.1% (v/v) formic acid.

### nLC-MS/MS and data analysis

Peptides derived from extracted and digested proteins were analyzed by nLC-MS/MS (Biqualys, Wageningen, Netherlands) as described earlier (Lu et al., 2011). MaxQuant v.1.3.0.5 with default settings for the Andromeda search engine (Cox and Mann, 2008) in the label free quantitation mode was used to analyze MS and MS/MS spectra, except that extra variable modifications were set as described before (Rupakula et al., 2013). A protein database of *D. restrictus* was generated from the whole genome sequence (Kruse et al., 2013) using the Artemis genome browser (release 15.0.0). Also, a contaminant database including sequences of common contaminants like trypsin, BSA and human keratins (Rutherford et al., 2000; Rupakula et al., 2013) was used. Further filtering and bioinformatics analysis was performed with Perseus software v. 1.3.0.4 as described before (Smaczniak et al., 2012). Also, protein groups with a logarithmic label-free quantitation (LFQ) intensity of zero for all treatments were deleted from the MaxQuant result table. Subsequently, remaining Log LFQ zero values were replaced by 5 (slightly below the lowest value measured) in order to make sensible ratio calculations possible. Students *T*-test was used to identify significant differences in the proteome when comparing logarithmic LFQ values obtained from two culture conditions.

## Results

Corrinoids are essential as a growth factor for *D. restrictus* (Holliger et al., 1998). The corrinoid present in the PCE reductive dehalogenase (PceA) of *D. restrictus* is presumably similar to the type added to the medium, i.e., cobalamin (Maillard et al., 2003). Detailed analysis of the genome of *D. restrictus* revealed a seemingly complete corrinoid biosynthesis pathway. Compared to other *Dehalobacter* genomes, however, a 101-bp fragment was found to be missing in the *cbiH* gene of *D. restrictus* (Kruse et al., 2013; Rupakula et al., 2013) (Figure S1). The present study aimed specifically at obtaining a broader understanding of the corrinoid metabolism in *D. restrictus*.

### Growth of *D. restrictus* under corrinoid-limiting conditions

The full corrinoid biosynthetic pathway was described earlier (Rupakula et al., 2013). A modified and extended version of it is depicted in Figure 1. Briefly, the pathway can be divided in two branches, namely the upper corrinoid biosynthesis (UCB) and the nucleotide loop assembly (NLA), which are connected at the level of ado-cobyric acid.

**Figure 2:**
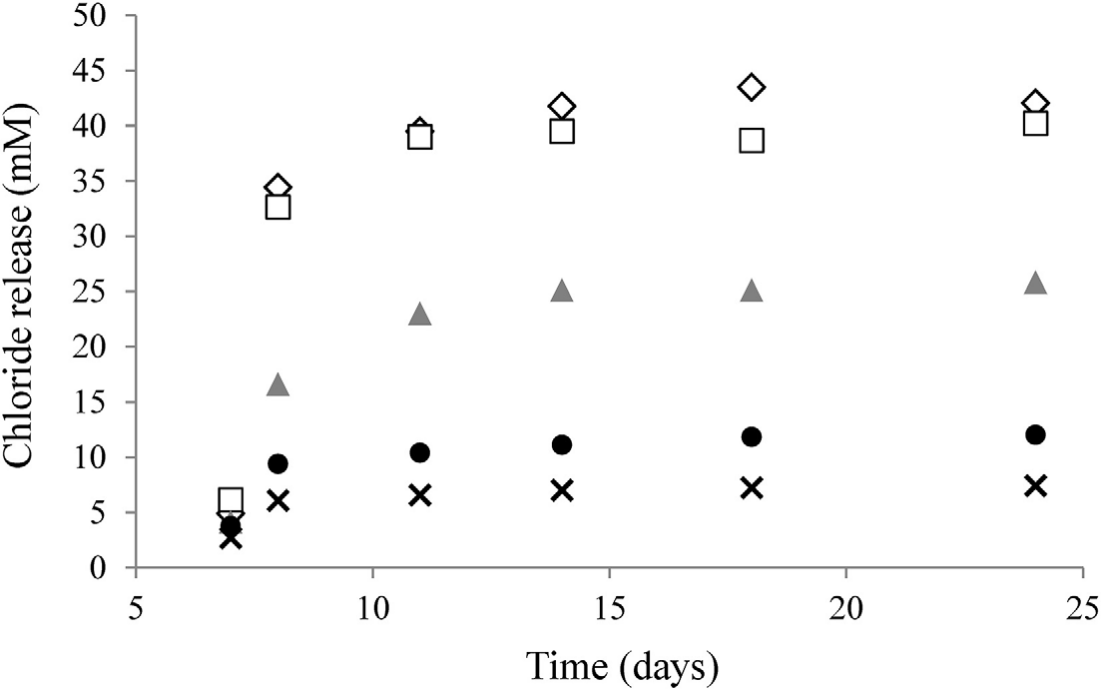
**Corrinoid starvation effect on PCE dechlorination by *D. restrictus* by lowering the initial concentration of corrinoid supplemented into the medium**. Corrinoid concentration: 250 μ g/L (white diamonds), 50 μ g/L (white squares), 10 μ g/L (gray triangles), 5 μ g/L (black circles) and 1 μ g/L (black crosses).

In the present study, batch cultures were cultivated with addition of 250 μg/L cyanocobalamin to the growth media. An experiment was performed to assess the effects of lowering the initial corrinoid concentration in the medium (250, 50, 10, 1 μg/L and no corrinoid) on dechlorination, which for this obligate OHRB is also a good estimation for growth (Figure 2) (Holliger et al., 1998). The extent of PCE dechlorination was the same in cultures provided with 50 or 250 μg/L corrinoid demonstrating that the former was enough to reach the maximum dechlorination capacity. In contrast, the chloride release was only half of the maximum in cultures supplemented with 10 μg/L corrinoid, implying that availability of corrinoids was a limiting factor. Further lowering the corrinoid concentration to 1 or 0 μg/L resulted in negligible levels of dechlorination, and therefore growth was assumed to be abolished in these cultures.

### Corrinoid metabolic gene arrangement in *D. restrictus*

The complete corrinoid biosynthetic and uptake pathway is genetically encoded in *D. restrictus*. These genes can be divided into four functional groups depending on the part of the pathway they encode for (Figure 1). The first group denoted as the upper corrinoid biosynthesis (UCB) pathway genes contains genes required to synthesize ado-cobyric acid. The second group consists of genes required for synthesis and the nucleotide loop assembly (NLA) of corrinoids, and the third functional group comprises the corrinoid salvaging (CS) pathway, i.e., genes involved in remodeling corrinoid intermediates salvaged from the environment into ado-cobyric acid (*cbiZ* gene family). The fourth group harbors both cobalt and corrinoid transporter encoding genes (CT).

**Figure 3:**
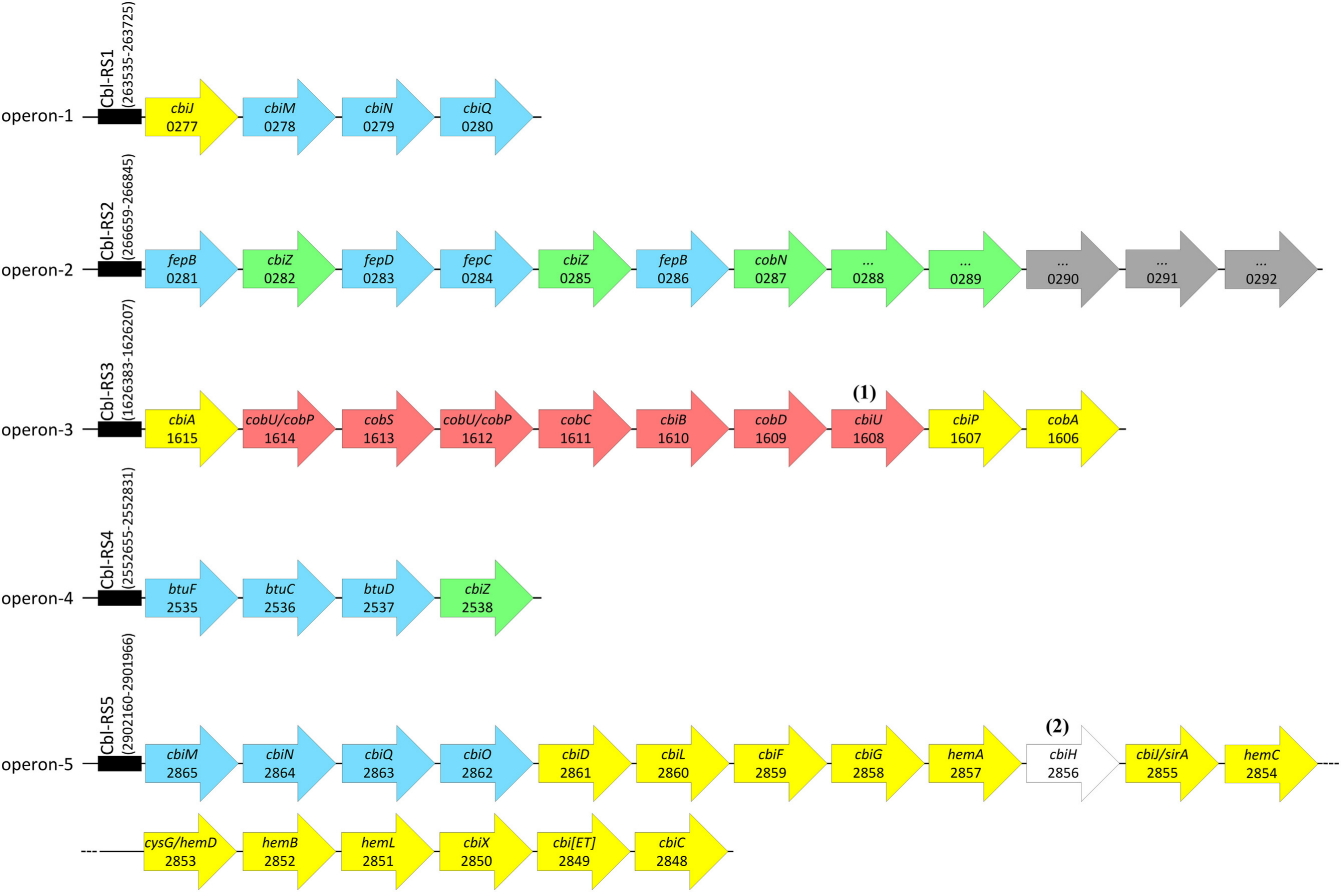
**Arrangement of genes associated with corrinoid biosynthesis and uptake in the genome of *D. restrictus***. Genes involved in corrinoid metabolism are grouped in five operons (operon-1 to -5) located in different places of the genome. All five operons are predicted to be regulated by cobalamin riboswitches (Cbl-RS1 to -5, indicated by a black box) located directly upstream of the first gene of each operon. Cbl-RS coordinates are given based on their identification by Rfam. Genes are depicted as arrows in colors corresponding to four distinct pathways (see also Figure 1): upper corrinoid biosynthesis (UCB, in yellow), nucleotide loop assembly (NLA, in red), cobalt and corrinoid transporters (in blue) and corrinoid salvage (in green). Gray arrows depict genes that have no clear function in corrinoid biosynthesis. Gene names and corresponding gene loci (with the Dehre_# prefix removed) are given when available. Notes: (1) *cbiU* was newly annotated as a possible L-threonine kinase encoding gene. (2) a 101-bp deletion makes *cbiH* non-functional in *D. restrictus*.

Most genes associated with corrinoid metabolism are arranged in the genome of *D. restrictus* in five gene clusters (referred to as operon-1 to -5), which are roughly organized according to the function they play in corrinoid biosynthesis (Figure 3). Most proteins involved in the UCB pathway are encoded in operon-5 with the exception of the three last steps that are catalyzed by the product of genes present in operon-3. This latter operon also codes for the enzymes involved in the NLA pathway. Within operon-3, the locus Dehre_1608 was initially annotated as a phosphoglycerate mutase, but shows also sequence similarity with archaeal-type homoserine kinase (with conserved domain TIGR02535) involved in the synthesis of threonine. Here, we propose it could act as an L-threonine kinase (in analogy to PduX in *Salmonella* (Fan and Bobik, 2008)), which might therefore be involved in the production of aminopropanol-phosphate. No *pduX* homolog could be identified in *D. restrictus*, suggesting that this function is fulfilled by the gene product of Dehre_1608. Hence, we propose to name it *cbiU*. Operon-1 contains a homolog of *cbiJ* (besides the *cbiJ*/*cysG* gene, Dehre_2855, present in the conserved biosynthesis operon-5), and a set of genes coding for the energy-coupling factor-type CbiMNQ cobalt transporter. An additional, albeit different *cbiMNQO* gene cluster is also present at the 5′-end of operon-5 together with the genes for the UCB pathway. Operon-2 harbors a combination of genes coding for transporters (with sequence similarity to FepBCD/BtuCDE ABC-type transporters) likely involved in corrinoid transport, the genes for two different salvaging enzyme (CbiZ) paralogues (Dehre_0282 and _0285), a gene cluster encoding the cobaltochelatase CobN (Dehre_0287), and several subunits of a magnesium chelatase complex. Finally, operon-4 contains a gene cluster coding for an ABC-type corrinoid transporter (BtuFCD) and another copy of *cbiZ* (Dehre_2538). Two additional genes potentially involved in corrinoid biosynthesis (*cobT*/Dehre_1488 and *cobB*/*cobQ*, Dehre_2360) are located elsewhere in the genome and not in one of the five operons.

### Comparative genomics of corrinoid operons in *dehalobacter* spp.

The genome of *D. restrictus* was compared with newly available genomes of *Dehalobacter* spp. strains DCA, CF, E1, FTH1, and UNSWDHB with regard to the organization of corrinoid operons (Table 1 and Table S1). Synteny maps for operon-1 and -2 (Figure S2), and for operon-3, -4, and -5 (Figures S3–S5, respectively) are given as Supplementary Material. Operon-1 is conserved in *D. restrictus* and *Dehalobacter* sp. E1 but absent in all other genomes. Operon-2, which is directly following operon-1 in *D. restrictus*, is lacking in all other *Dehalobacter* spp. for which genome sequences are available to date. However, a detailed analysis of *Dehalobacter* sp. E1 suggests that operon-2 was lost in that strain as the sequence conservation with *D. restrictus* is extended slightly beyond operon-1 but is readily interrupted within the homolog of Dehre_0281 (the first gene of operon-2 in *D. restrictus*). This deletion in strain E1 includes all of the remaining genes operon-2 and beyond, as a 5′-truncated version of Dehre_0297 is again found in strain E1 (Figure S2, panel C). *D. restrictus* operon-3 to -5 are fully conserved in all *Dehalobacter* spp. with the exception of another deletion in the proximal region of operon-5 in strain E1 (Figure S5).

**Table 1:** **Comparative genomics of corrinoid operons in *Dehalobacter* spp**.

	***Dehalobacter restrictus***	***Dehalobacter* sp. E1**	***Dehalobacter* sp. DCA**	***Dehalobacter* sp. CF**	***Dehalobacter* sp. FTH1**	***Dehalobacter* sp. UNSWDHB**
Operon–1	+	+	−	−	−	−
Operon–2	+	−	−	−	−	−
Operon–3	+	+	+	+	+	+
Operon–4	+	+	+	+	+	+
Operon–5	+	Partial	+	+	+	+
*cbiH*	Deletion	Intact	Intact	Intact	Intact	Intact

In *D. hafniense* in contrast, the corrinoid biosynthesis genes are organized in two operons. The major operon (corresponding to DSY4057-4072 in *D. hafniense* strain Y51) encodes proteins of the UCB pathway and part of the NLA pathway, while a 3-gene operon (DSY2114-2116) encodes for the remaining NLA proteins (Nonaka et al., 2006; Choudhary et al., 2013). Some proteins encoded by *D. restrictus* operon-2 have their counterpart in other OHRB. For example, corrinoid transporters are present in most OHRB, however, corrinoid producers such as *D. hafniense* and *Sulfurospirillum multivorans* do not harbor any *cbiZ* homologous gene. In *D. mccartyi* in contrast, multiple *cbiZ* genes are present in the genomes (Figure S6).

All proteins encoded in operon-2 of *D. restrictus* share between 50 and 77% sequence identity with homologous proteins present in the non-dechlorinating *Firmicute Acetobacterium woodii*. A high level of genetic synteny was further identified between operon-2 of *D. restrictus* and a part of the genome of *A. woodii* (GenBank NC_016894.1, Poehlein et al., 2012) (Figure S7). Significant sequence similarity of individual proteins of operon-2 was mostly found with homologs of some other members of *Clostridia* and a few *δ-Proteobacteria* (data not shown).

### Identification of cobalamin riboswitches in *D. restrictus*

Upstream of each of the five corrinoid biosynthesis-related operons in *D. restrictus* a distinct cobalamin riboswitch (Cbl-RS) was identified using Rfam. These five sequences were manually refined in a similar way as done previously for the Cbl-RS sequences of *D. hafniense* (Choudhary et al., 2013). The alignment of structurally conserved regions of *D. restrictus* riboswitches (Cbl-RS01 to -RS05) was compared to *E. coli btuB* Cbl-RS (Figure S8). In contrast to *E. coli* Cbl-RS, which is regulated at the level of translation (Nahvi et al., 2004), all five *D. restrictus* Cbl-RS sequences end with a predicted transcriptional terminator, suggesting that the regulation operates at the level of transcription.

### Transcriptional analysis of corrinoid biosynthesis operons in *D. restrictus*

**Figure 4:**
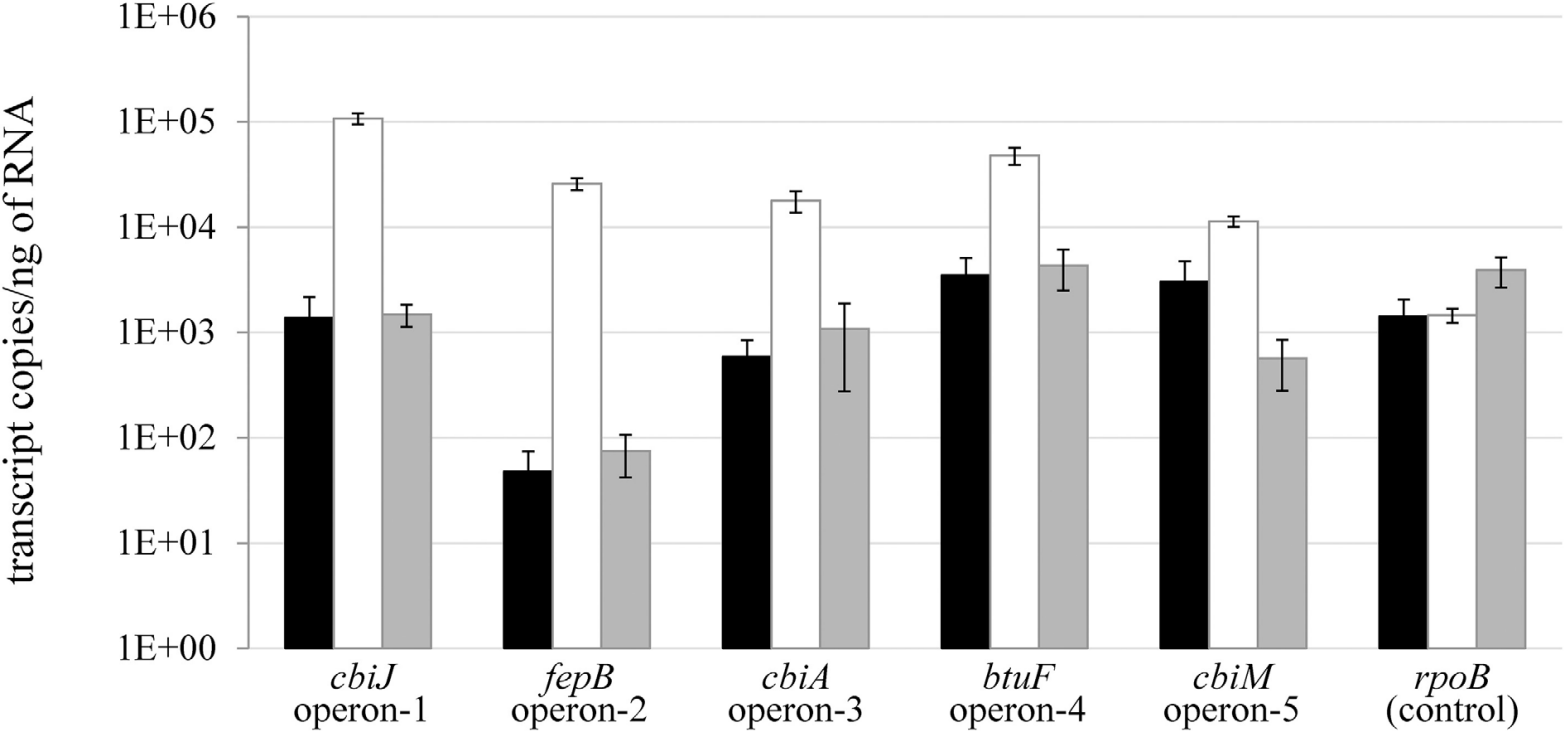
**Transcriptional analysis of cobalamin riboswitch-dependent genes in *D. restrictus***. The transcription of the genes located directly downstream of the cobalamin riboswitches in operon-1 to -5 was analyzed. The housekeeping gene *rpoB* was used as unregulated control. The black bars indicate the transcriptional level under corrinoid standard conditions (250 μ g/L), the white bars under corrinoid starvation conditions (10 μ g/L), and the gray bars show the transcriptional level 2 h after replenishment of the latter cultures with 250 μ g/L cyanocobalamin. The data show the mean of triplicate cultures with standard deviation.

The transcription of genes located directly downstream of the Cbl-RS in *D. restrictus* was analyzed for cells cultivated in the presence of high (250 μ g/L) and low (10 μ g/L) corrinoid concentration, and after corrinoid replenishment from low to high concentrations (Figure 4). Quantitative PCR was applied on complementary DNA targeting the first gene located directly downstream of each cobalamin riboswitch. Analysis of corrinoid-starved *D. restrictus* RNA revealed a higher transcription level of these genes, confirming an active regulation of the respective riboswitches at transcriptional level. Two hours after corrinoid replenishment, transcription of all selected genes was again repressed to the same level as observed under high corrinoid concentration. However, individual responses were significantly different. Indeed, the most pronounced effect was observed for two genes, namely Dehre_0277 (73-fold repression) and _0281 (346-fold), corresponding to the first genes in operon-1 and -2 in *D. restrictus*, respectively.

### Proteome analysis of corrinoid starvation in *D. restrictus*

Comparative whole-proteome analysis was done on *D. restrictus* PER-K23 cells cultivated in the presence of 250 (high), 50 (mid) or 10 (low) μg/L cyanocobalamin. A total of 1195 proteins were detected, corresponding to 42% of the predicted 2826 proteins encoded on the genome (Kruse et al., 2013). The majority of the detected proteins (1175) were identified in cells from all the tested cyanocobalamin concentrations (Table S4). Normalized LFQ protein intensities were used to compare relative abundances of proteins between different cyanocobalamin treatments. A minimal change of 3-fold in LFQ protein intensity was considered throughout the study. The abundance of 44 proteins showed significant difference (*P* < 0.01) between high (250 μg/L) and low (10 μg/L) corrinoid concentration, and the relative abundance of another 29 proteins showed more than 10-fold changes, albeit not significant due to high variation between triplicates (Figure S9). The results for protein abundance ratios between high and mid, and between mid and low are in the same range (see Table S3).

Proteins associated with cobalamin biosynthesis were further analyzed. A complete *de novo* corrinoid biosynthesis pathway was predicted in the genome of *D. restrictus* starting from glutamyl-tRNA to cobalamin (Figure 1) (Kruse et al., 2013; Rupakula et al., 2013). All proteins required for biosynthesis of ado-cobyric acid from cobalt-precorrin 5B were identified in proteomic data including CbiD (cobalamin biosynthesis protein, Dehre_2861), an alternative CbiJ (precorrin-6x reductase, Dehre_0277) and Cbi[ET] (precorrin-6Y methyltransferase, Dehre_2849), which were not dectected in a previously analyzed proteome from *D. restrictus* (Rupakula et al., 2013). However, CbiH (precorrin-3B C17-methyltransferase, Dehre_2856), CbiG (cobalamin biosynthesis protein, Dehre_2858) and CbiJ/SirA (precorrin-6x reductase, Dehre_2855) belonging to the UCB pathway and CobS (cobalamin 5′-phosphate synthase, Dehre_1613) of the NLA pathway were not found in the current proteome analysis. The lack of CbiH in the proteome is in line with the observation of a 101-bp deletion in *cbiH* likely leading to a non-functional gene (Figure S1), thus likely to explain why *D. restrictus* requires exogenous corrinoids supply to the growth medium.

Previously, the presence of one ABC-type cobalt transporter (Dehre_0850-0852) and two energy-coupling factor-type cobalt transporters (Dehre_0278-0280 and Dehre_2862-2865) was predicted in the genomic study of *D. restrictus* (Rupakula et al., 2013). Here, we identified CbiM (Dehre_0278), CbiQ (Dehre_0280), CbiO (Dehre_2862), and CbiN (Dehre_2864) in the proteome dataset obtained in this study (Table S4).

**Figure 5:**
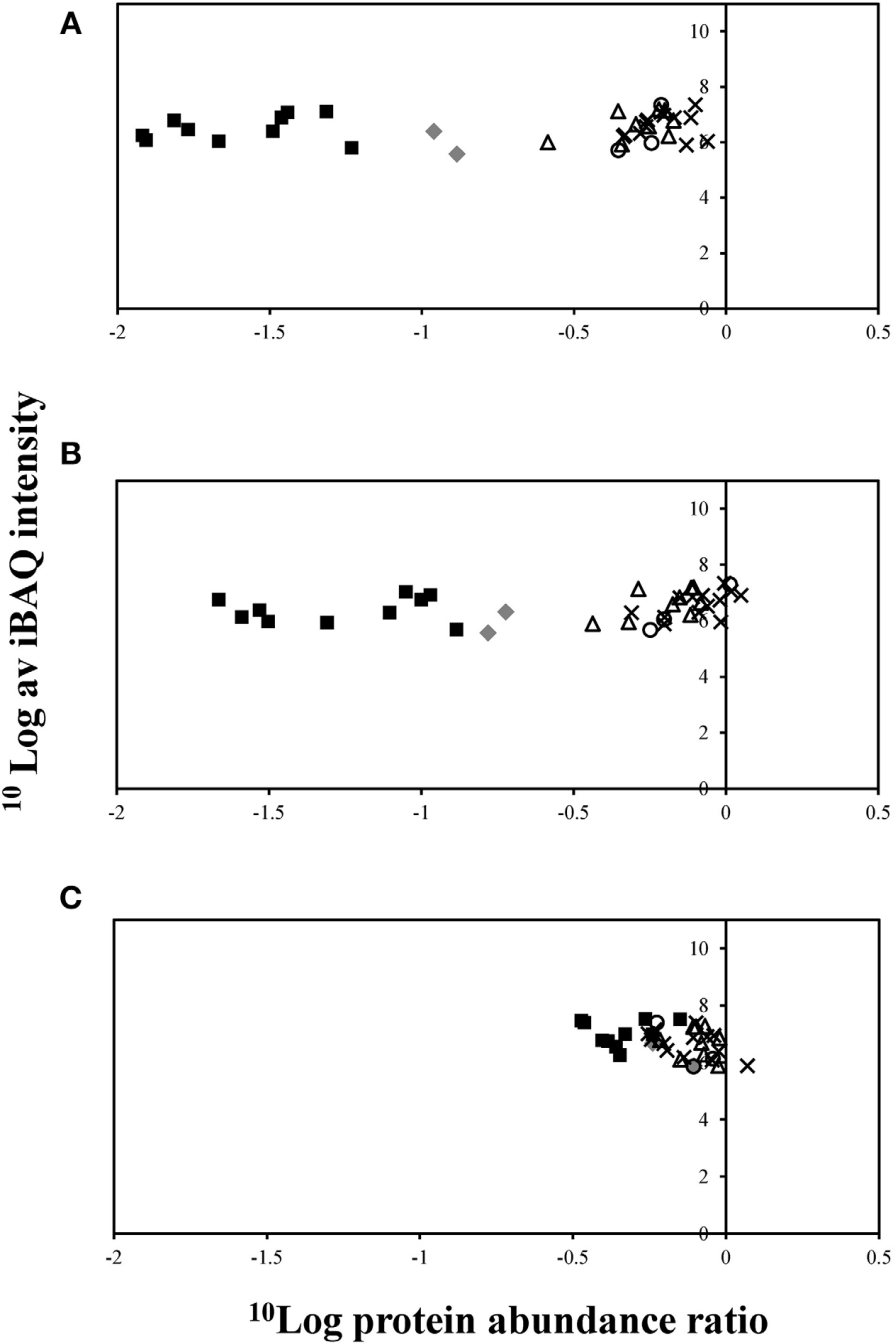
**Proteins associated with corrinoid biosynthesis and uptake, extracted from *D. restrictus* cells cultivated in the presence of different cyanocobalamin concentrations. (A)** Proteomics analysis of cells cultivated in the presence of high (250 μg/L) vs. low (10 μg/L) corrinoid concentrations; **(B)** high (250 μg/L) vs. mid (50 μg/L) corrinoid concentrations; **(C)** mid (50 μg/L) vs. low (10 μg/L) corrinoid concentrations. Proteins encoded by corrinoid operon-1 (gray diamonds), operon-2 (black squares), operon-3 (white triangles), operon-4 (white circles), and operon-5 (black crosses) are shown. Logarithmic average of iBAQ (Intensity based absolute quantitation) value is plotted against the log value of protein abundance ratio based on LFQ value. CbiM (Dehre_0278) was left out because it could not be identified in two replicates of cultures under low corrinoid concentration.

Relative abundance of proteins associated with corrinoid biosynthesis and salvaging pathways was further analyzed. Interestingly, nearly all proteins associated with corrinoid biosynthesis and salvaging pathways were up-regulated under corrinoid limiting growth conditions (Figure 5 and Table S5). As expected the overall largest change in the abundance of proteins related to corrinoid biosynthesis and salvaging pathways was observed when comparing the proteome of cells cultivated at high vs. low concentration of cyanocobalamin (Figure 5A). The corrinoid metabolism differed more strongly when comparing cells cultivated in the presence of high vs. mid than mid vs. low concentrations (Figures 5B,C).

Proteins encoded by operon-2 showed the largest change in protein abundance ratios with on average 46-fold up-regulation when comparing cells cultivated at low vs. high corrinoid concentrations (Figure 5A). Operon-2 encodes proteins predicted to be involved in corrinoid salvaging or corrinoid transport. Among these are two predicted CbiZ proteins, amidohydrolases required for salvaging the corrinoid precursor cobinamide, which were up-regulated 80-fold (Dehre_0285) and 58-fold (Dehre_0282) under corrinoid starvation, respectively (Table S4). Furthermore, proteins encoded in operon-1 including an energy-coupling factor-type cobalt transporter (Dehre_0278-0280) which is likely to be involved in the cobalt uptake process, and a precorrin-6x reductase (Dehre_0277), were on average 8-fold up-regulated when comparing cells cultivated in the presence of low vs. high corrinoid concentrations. Fewer changes were found for proteins encoded by the three remaining corrinoid-related operons under the different corrinoid conditions.

## Discussion

### Corrinoid biosynthesis of *D. restrictus* and other OHRB

In the present study, ≥50 μg/L of cyanocobalamin was required for *D. restrictus* to reach its maximum PCE dechlorination, in line with previous observation that this organism depends on externally supplemented corrinoids (Holliger et al., 1998). The genome of *D. restrictus* encodes a complete set of corrinoid biosynthesis genes, with the exception of a non-functional *cbiH* gene, suggesting that tetrapyrrole ring contraction does not occur here and represents a dead-end in the biosynthesis pathway. Comparative genomic analysis among other *Dehalobacter* spp. revealed that an intact *cbiH* gene is present in all other genomes. However, little is known about the capacity of other members of this genus to *de novo* synthesize corrinoids. Indeed they only have been studied under growth conditions with external addition of cyanocobalamin or in co-cultures (Grostern and Edwards, 2006; Yoshida et al., 2009; Grostern et al., 2010; Maphosa et al., 2012; Deshpande et al., 2013). Similarly, strains of *D. mccartyi*, which are also obligate OHRB, are corrinoid-auxotroph (Löffler et al., 2013). Unlike *D. restrictus*, the corrinoid auxotrophy in *D. mccartyi* strains is due to the lack of the complete biosynthetic pathway. Instead they rely on uptake of extracellular corrinoids via the salvaging pathway and on remodeling of incomplete or non-functional corrinoids in the presence of appropriate free lower ligands, among which 5,6-dimethylbenzimidazole plays a key role (Yan et al., 2012, 2013; Yi et al., 2012; Schipp et al., 2013; Men et al., 2014a). Interestingly, most facultative OHRB such as *S. multivorans* (Kräutler et al., 2003), *D. hafniense* (Nonaka et al., 2006; Choudhary et al., 2013) or *G. lovleyi* (Wagner et al., 2012) are capable of *de novo* biosynthesis of corrinoids.

The genome of *D. restrictus* encodes five well-organized operons containing most of the corrinoid biosynthesis-associated genes. Comparing the genomes of the sequenced *Dehalobacter* spp. revealed that *D. restrictus* harbors an extra set of genes (operon-2) coding for putative corrinoid transporters and salvaging enzymes (CbiZ and cobaltochelatases), suggesting an augmented capacity for corrinoid uptake and remodeling compared to other *Dehalobacter* spp. The importance of *cbiZ* genes in remodeling corrinoids has already been demonstrated for *D. mccartyi* (Kube et al., 2005; Seshadri et al., 2005; Men et al., 2014a). The role of operon-2 in *D. restrictus* was evidenced by the significant up-regulation of the corresponding enzymes when corrinoid concentration in the medium was lowered. This result clearly showed that *D. restrictus* has developed a particular strategy to cope, at least partially, with its lack of corrinoid biosynthesis under unfavorable corrinoid conditions. The presence of additional *cbiZ* genes in operon-2 raises the questions of the functional redundancy *vs*. specificity of multiple CbiZ proteins within a single strain, and of the origin of the additional *cbiZ* genes present in *D. restrictus*. While *in vitro* biochemical investigations would be required to answer the first question, a detailed analysis of CbiZ sequence homology (Figure S6) revealed that the two additional CbiZ proteins in *D. restrictus* show a high level of sequence identity with CbiZ homologs present in *A. woodii*, a corrinoid-producing bacterium (Stupperich et al., 1988), which has been well-characterized for the Wood-Ljungdahl pathway that also requires corrinoids as an essential cofactor (Ragsdale and Pierce, 2008). The high degree of genetic synteny identified between the operon-2 of *D. restrictus* and *A. woodii* suggests that *D. restrictus*, but not the other members of the *Dehalobacter* genus, most probably acquired operon-2 by horizontal gene transfer and successfully exploited this operon to partially alleviate the loss of a functional *cbiH* gene.

### Effect of corrinoid starvation on *D. restrictus* metabolism

Reduction of cyanocobalamin amendment in the growth medium strongly inhibited PCE dechlorination by *D. restrictus*. It also had a profound effect on *D. restrictus* corrinoid metabolism both at the level of transcription and at the proteome level. While changing from high (250 μg/L) corrinoid concentration to an intermediate concentration (50 μg/L), *D. restrictus* responded by up-regulating proteins associated with corrinoid transport and salvaging pathways encoded in operon-1 and -2, allowing the strain to reach the same PCE dechlorination level as observed during high corrinoid concentration. Decreasing the corrinoid concentration even further to 10 μg/L showed, however, that, while the extent of PCE dechlorination was strongly affected, the amount of corrinoid-associated proteins did not notably change when compared to cells cultivated in the presence of 50 μg/L corrinoid. This indicates that at corrinoid concentrations as low as 10 μg/L, *D. restrictus* was not able to compensate the lack of externally provided corrinoids by increased corrinoid transport and salvaging.

The presence of cobalamin riboswitches directly upstream of the five corrinoid operons in *D. restrictus* already suggested an active repression at the level of transcription by cyanocobalamin. Similar to transcriptional studies on *D. mccartyi* (Johnson et al., 2009) and *D. hafniense* (Choudhary et al., 2013), the cobalamin riboswitches of *D. restrictus* responded to addition of excess cyanocobalamin, and the level of repression of the gene located directly downstream of the riboswitches correlated well with the proteomic data, showing the strongest effect for *cbiJ* (Dehre_0277, operon-1) and for *fepB* (Dehre_0281, operon-2). The sequence of individual cobalamin riboswitches is likely responsible for their differential responsiveness toward cobalamin concentration, as both their affinity to cobalamin and the strength which the expression platform exerts on transcriptional repression are sequence dependent. Such effects have already been shown for a few cobalamin riboswitches in *D. hafniense* (Choudhary et al., 2013).

*D. mccartyi* strain 195, another corrinoid-auxotroph, requires a concentration of 25 μg/L cyanocobalamin to support optimal TCE dechlorination rates and growth yield (He et al., 2007), a value that is similar to what was observed for *D. restrictus*. Therefore, and in addition to the ecogenomic biomarkers defined by Maphosa et al. (2010), one could consider the physiological threshold of corrinoid concentration as a possible diagnostic tool to delineate the reductive dechlorination potential by corrinoid-auxotrophic OHRB in anaerobic environments. Meanwhile, the production of the PCE reductive dehalogenase (PceA, Dehre_2398) in *D. restrictus* showed no significant change under different corrinoid concentrations, which strongly suggests that the amount of available corrinoid and not of the apo-enzyme represents the main limiting factor for PCE dechlorination.

Taken altogether, our results support the hypothesis that, besides the partial deletion of *cbiH* in *D. restrictus* (Kruse et al., 2013; Rupakula et al., 2013), which already represents a crucial checkpoint in the corrinoid biosynthesis pathway, the energetic cost of *de novo* corrinoid biosynthesis might explain why *D. restrictus* has developed enhanced corrinoid transport and salvaging strategies. *D. restrictus* corrinoid metabolism represents an intermediate situation between the true corrinoid-auxotrophic and obligate organohalide-respiring *D. mccartyi*, which lacks the corrinoid biosynthesis pathway completely (He et al., 2007; Men et al., 2012, 2014a; Yan et al., 2012), and the facultative OHRB able to produce corrinoids *de novo*.

## Author contribution

Aamani Rupakula and Yue Lu performed the experiments, analyzed the data and wrote the manuscript. Thomas Kruse analyzed the data and revised the manuscript. Sjef Boeren performed the experiments and revised the manuscript. Christof Holliger and Hauke Smidt revised the manuscript. Julien Maillard designed the work, analyzed the data and wrote the manuscript.

### Conflict of interest statement

The authors declare that the research was conducted in the absence of any commercial or financial relationships that could be construed as a potential conflict of interest.

## Abbreviations

Cbl-RS: cobalamin riboswitch
CS: corrinoid salvaging
CT: corrinoid transporter
LFQ: label free quantitation
NLA: nucleotide loop assembly
OHR: organohalide respiration
OHRB: organohalide respiring bacteria
PCE: tetrachloroethene (perchloroethylene)
RDase: reductive dehalogenase
TCE: trichloroethene
UCB: upper corrinoid biosynthesis.
