# Supplementary material for: Functional genomics of corrinoid starvation in the organohalide-respiring bacterium Dehalobacter restrictus strain PER-K23
Source: Front Microbiol. 2015 Jan 6;5:751. doi: 10.3389/fmicb.2014.00751 (PMC4285132; doi:10.3389/fmicb.2014.00751)
Supplement: Supplementary file 2 [file Table2.PDF]

Supplementary material

To the article ‘Functional genomics of corrinoid starvation in the organohalide-respiring bacterium *Dehalobacter restrictus* strain PER-K23’ by A. Rupakula, Y. Lu, T. Kruse, S. Boeren, C. Holliger, H. Smidt and J. Maillard.

Table S2. Oligonucleotides and plasmids used in this study

A. Oligonucleotides used in this study

Primer	Primer sequence (5’-3’)	Target gene	*D. restrictus* locus	Amplicon size (bp)
DRE1-f	TCATCCTTCTGGGAGAAACG	*cbiJ*	Dehre_0277	274
DRE1-r	GCTTCCTCCCAATTACATGC			
DRE2-f	TACCGGAGATTACGGACTGC	*fepB*	Dehre_0281	263
DRE2-r	ACACCTTGCCATCGTAGACC			
DRE3-f	CCATTTTAGACCGGACAACG	*cbiA*	Dehre_1615	235
DRE3-r	GGATTCTCTGGCCTGTATGG			
DRE4-f	TTGAAGAGCTGAAGGTGACG	*btuF*	Dehre_2535	173
DRE4-r	GGCGATGTCTTTGAGTTTGG			
DRE5-f	CTGCGGGTTGGTGTATTACC	*cbiM*	Dehre_2865	215
DRE5-r	GAACAAGATAGCGCCAAAGC			
RPOB-f	GGAAAATCCGTTCTTTATGACG	*rpoB*	Dehre_0463	276
RPOB-r	TACCACATCATCGGACTTAACG			

B. Plasmids used in this study

Plasmid	Description	Reference
pGEM-T Easy	Vector for direct cloning of PCR products	Promega
pDRE1	Fragment of *cbiJ* (Dehre_0277) cloned into pGEM-T Easy	This study
pDRE2	Fragment of *fepB* (Dehre_0281) cloned into pGEM-T Easy	This study
pDRE3	Fragment of *cbiA* (Dehre_1615) cloned into pGEM-T Easy	This study
pDRE4	Fragment of *btuF* (Dehre_2535) cloned into pGEM-T Easy	This study
pDRE5	Fragment of *cbiM* (Dehre_2865) cloned into pGEM-T Easy	This study
pRPOB	Fragment of *rpoB* (Dehre_0463) cloned into pGEM-T Easy	This study
