# Supplementary material for: Functional genomics of corrinoid starvation in the organohalide-respiring bacterium Dehalobacter restrictus strain PER-K23
Source: Front Microbiol. 2015 Jan 6;5:751. doi: 10.3389/fmicb.2014.00751 (PMC4285132; doi:10.3389/fmicb.2014.00751)
Supplement: Supplementary file 3 [file Table3.PDF]

Supplementary material

To the article 'Functional genomics of corrinoid starvation in the organohalide-respiring bacterium *Dehalobacter restrictus* strain PER-K23' by A. Rupakula, Y. Lu, T. Kruse, S. Boeren, C. Holliger, H. Smidt and J. Maillard.

Table S3. Summary of proteome analysis

	**Cobalamin concentration ratios**		
	**high/mid**	**mid/low**	**high/low**
> 3-fold up	24	43	36
> 3-fold down	68	39	68
Total	92	82	104
up (p<0.01)	27	14	34
down (p<0.01)	11	33	10
Total	38	47	44
