# Supplementary material for: Functional genomics of corrinoid starvation in the organohalide-respiring bacterium Dehalobacter restrictus strain PER-K23
Source: Front Microbiol. 2015 Jan 6;5:751. doi: 10.3389/fmicb.2014.00751 (PMC4285132; doi:10.3389/fmicb.2014.00751)
Supplement: Supplementary file 6 [file Image1.PDF]

Supplementary material

To the article ‘Functional genomics of corrinoid starvation in the organohalide-respiring bacterium *Dehalobacter restrictus* strain PER-K23’ by A. Rupakula, Y. Lu, T. Kruse, S. Boeren, C. Holliger, H. Smidt and J. Maillard.

```
Dre_cbiH       1 ATGAAAATATATGTCGTAGGATTAGGGCCAGGCGGACCGGAACAAATGACAAACCGTGCA
Dhb-CF_cbiH    1 ATGAAAATATATGTCGTGGGATTAGGACCAGGCGGACCGGAGCAAATGACAAACCGCGCG

Dre_cbiH      61 CAGGAAGCACTTAGAAAAAGTGATATTTTAGTCGGGTATACGCTTTATATCGATTTAATC
Dhb-CF_cbiH   61 CAGGAAGCGCTTAAGGCCAGTGATATTTTAGTCGGATATACGCTTTATATCGATTTAATC

Dre_cbiH     121 AAGGATCAGTTCCCGGATAAAAAGTTTATTGCTACGCCGATGAAGAAAGAGGTAGATCGC
Dhb-CF_cbiH  121 AAGGATCAGTTTCCAGATAAAAAGTTAATTGCTACGCCGATGAAGAAAGAGGTGGATCGC

Dre_cbiH     181 TGCAGGACCGCGGTTGAAAAGGCGCTGGAAGGCGCAACGGTTGCTGTGGTCTCGAGCGGG
Dhb-CF_cbiH  181 TGCAGGACCGCGGTTGAAAAGGCGCTGGAAGGCGCAACGGTTGCTGTGGTCTCGAGCGGG

Dre_cbiH     241 GATGCCGGAGTTTATGGCATGGCGGGAATCGTACTGGAGCTTGCAGAGCCGTATCCTTTC
Dhb-CF_cbiH  241 GATGCCGGAGTCTATGGCATGGCAGGAATCGTGCTGGAGCTTGCAGAGCCATATCCTTTC

Dre_cbiH     301 CTTGAGGTCGAAGTGATACCAGGAATCACAGCGGCCTGCAGCGG----------------
Dhb-CF_cbiH  301 CTCGAGGTCGAAGTGATACCAGGAATTACAGCAGCCTGCAGCGGTGCAGCTGTTCTCGGA

Dre_cbiH     345 ------------------------------------------------------------
Dhb-CF_cbiH  361 GCGCCACTCATTCATGACTTTGCAGTCATCAGCCTCAGTGATCTATTAACGCCGTGGGAG

Dre_cbiH     345 -------------------------CAGCCCAGGGAGACTTTGTCATTGTACTATACAAC
Dhb-CF_cbiH  421 AAGATTGAGGCTAGGCTTGCAGCAGCAGCTCAGGGAGACTTTGTCATTGTGCTCTATAAT

Dre_cbiH     380 CCATCGAGTATCAAAAGGCACGATTATTTGAAAAAGGCTTGCGATATTGTGATGCGCCAT
Dhb-CF_cbiH  481 CCATCGAGCATGAAAAGACACGATTATCTGAAAAGGGCCTGCGATATTGTGATGCGTCAT

Dre_cbiH     440 CAGGATGCTGACCTGCCGGCGGGCATTGTCCGCAGCATTGGCAGAGAGGGCGAGAATTAT
Dhb-CF_cbiH  541 CAGGATGCTAACTTGCCGGCGGGCATTGTCCGCAGCATCGGTAGAGAGGGCGAGAATTAT

Dre_cbiH     500 GAAATTGTCTCCAGCCTGACTGAATTGAGAGAAAAGAAAGTTGATATGTTTACGACTGTC
Dhb-CF_cbiH  601 GAGATTGTTTCCAGTCTGACTGAATTGAGAGAAAAGAAAGTTGATATGTTTACGACTGTC

Dre_cbiH     560 ATCATCGGGAATTCACAGACAAGGGTAATCAACAACAAACTGGTGACACCGAGAGGATAT
Dhb-CF_cbiH  661 ATTATTGGGAATTCACAGACAAGGATCATTAATAACAAACTGGTAACGCCGAGAGGATAT

Dre_cbiH     620 AAGAAATGA
Dhb-CF_cbiH  721 AAGAAATGA
```

Figure S1. Sequence alignment of *cbiH* genes of *D. restrictus* (*Dre*) and *Dehalobacter* sp. CF (*Dhb*-CF). A 101-bp deletion in *D. restrictus cbiH* gene created a frame-shift which terminates the corresponding polypeptide chain earlier (stop codon in red).
