# Supplementary material for: Functional genomics of corrinoid starvation in the organohalide-respiring bacterium Dehalobacter restrictus strain PER-K23
Source: Front Microbiol. 2015 Jan 6;5:751. doi: 10.3389/fmicb.2014.00751 (PMC4285132; doi:10.3389/fmicb.2014.00751)
Supplement: Supplementary file 7 [file Image2.PDF]

Supplementary material

To the article 'Functional genomics of corrinoid starvation in the organohalide-respiring bacterium *Dehalobacter restrictus* strain PER-K23' by A. Rupakula, Y. Lu, T. Kruse, S. Boeren, C. Holliger, H. Smidt and J. Maillard.

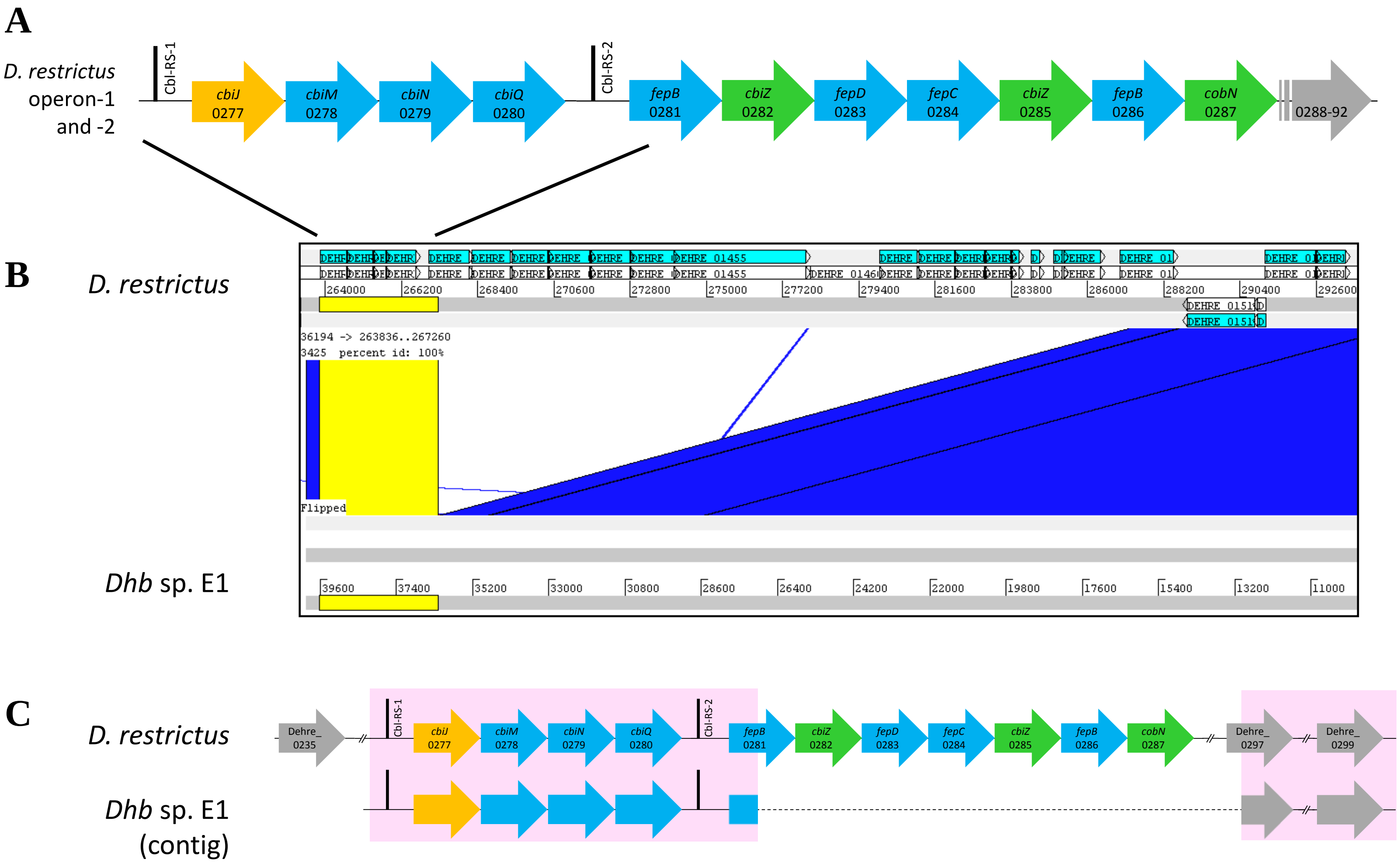

Figure S2. Synteny map of corrinoid operon-1 and -2 in *D. restrictus* and *Dehalobacter* sp. E1. (**A**) The gene arrays of corrinoid operon-1 and -2 of *D. restrictus* are preceded by their respective cobalamin riboswitches. (**B**). The yellow box in the synteny map indicates a nearly complete conservation of the genetic structure of operon-1 including the beginning of operon-2. The blue box indicates the conservation of genes beyond operon-2. Note the deletion in *Dehalobacter* sp. E1. (**C**) Alignment of the gene arrays of operon-1 and -2 in *D. restrictus* and *Dehalobacter* sp. E1.
