# Supplementary material for: Functional genomics of corrinoid starvation in the organohalide-respiring bacterium Dehalobacter restrictus strain PER-K23
Source: Front Microbiol. 2015 Jan 6;5:751. doi: 10.3389/fmicb.2014.00751 (PMC4285132; doi:10.3389/fmicb.2014.00751)
Supplement: Supplementary file 11 [file Image6.PDF]

**Supplementary material**

To the article 'Functional genomics of corrinoid starvation in the organohalide-respiring bacterium *Dehalobacter restrictus* strain PER-K23' by A. Rupakula, Y. Lu, T. Kruse, S. Boeren, C. Holliger, H. Smidt and J. Maillard.

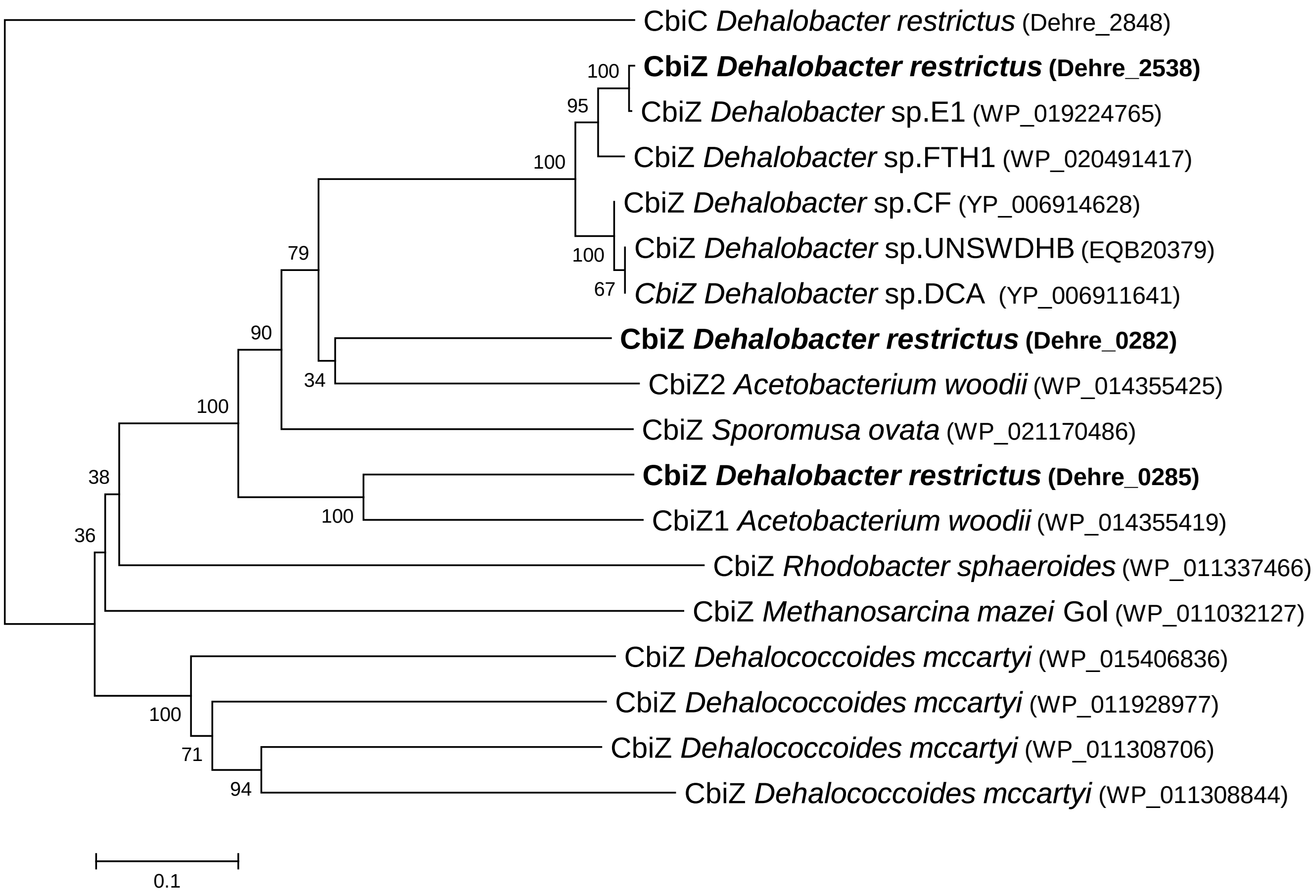


**Figure S6**. Maximal likelihood sequence analysis of CbiZ proteins. The three CbiZ proteins of *D. restrictus* (in bold) were aligned with ClustalX and compared to CbiZ proteins of other *Dehalobacter* spp. and of well-characterized bacteria either producing corrinoid (from the genera *Acetobacterium*, *Sporomusa*, *Rhodobacter*, *Methanosarcina*) or known to salvage it (*Dehalococcoides mccartyi*). CbiC of *D. restrictus* was used to root the tree. Sequence reference numbers are given in brackets.
