# Supplementary material for: Functional genomics of corrinoid starvation in the organohalide-respiring bacterium Dehalobacter restrictus strain PER-K23
Source: Front Microbiol. 2015 Jan 6;5:751. doi: 10.3389/fmicb.2014.00751 (PMC4285132; doi:10.3389/fmicb.2014.00751)
Supplement: Supplementary file 12 [file Image7.PDF]

Supplementary material

To the article 'Functional genomics of corrinoid starvation in the organohalide-respiring bacterium *Dehalobacter restrictus* strain PER-K23' by A. Rupakula, Y. Lu, T. Kruse, S. Boeren, C. Holliger, H. Smidt and J. Maillard.

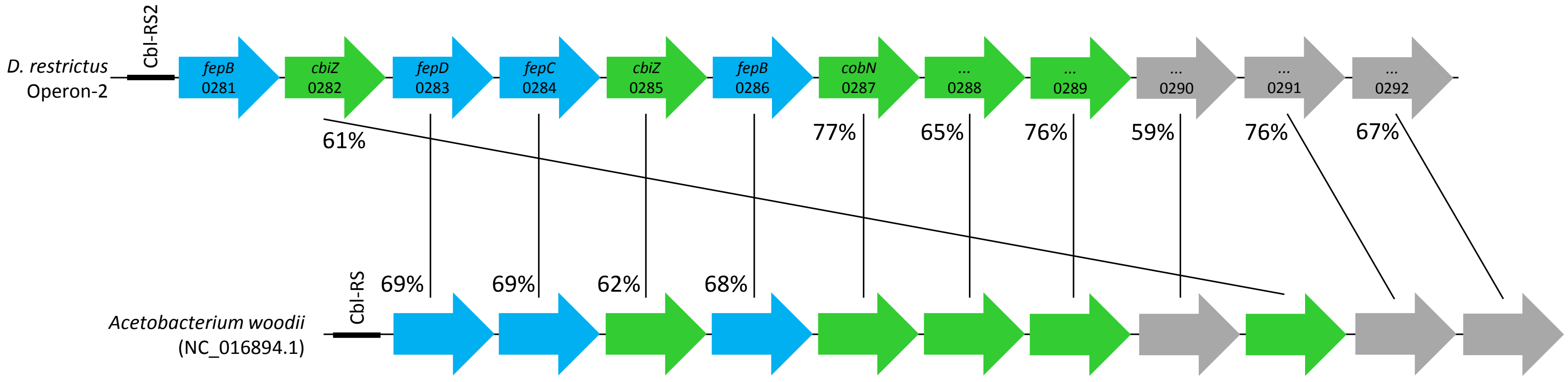

Figure S7. Synteny map of *D. restrictus* corrinoid operon-2 with a region of the genome of *Acetobacterium woodii* (DSM 1030). A high level of sequence identity was calculated for the product of all depicted genes. With the exception of one single gene (*cbiZ*, corresponding to Dehre_0282), the genetic organization is fully conserved between *D. restrictus* and *A. woodii*. The first gene in operon-2 in *D. restrictus* (Dehre_0281) seems to be the result of a gene duplication as it is missing in *A. woodii*. Finally, a cobalamin riboswitch was also identified upstream of the gene cluster in *A. woodii*. Blue and green arrows depict genes whose product is involved in corrinoid transport and corrinoid salvaging, respectively. Grey arrows represent genes not associated with corrinoid metabolism.
