# Supplementary material for: Functional genomics of corrinoid starvation in the organohalide-respiring bacterium Dehalobacter restrictus strain PER-K23
Source: Front Microbiol. 2015 Jan 6;5:751. doi: 10.3389/fmicb.2014.00751 (PMC4285132; doi:10.3389/fmicb.2014.00751)
Supplement: Supplementary file 13 [file Image8.PDF]

Supplementary material

To the article ‘Functional genomics of corrinoid starvation in the organohalide-respiring bacterium *Dehalobacter restrictus* strain PER-K23’ by A. Rupakula, Y. Lu, T. Kruse, S. Boeren, C. Holliger, H. Smidt and J. Maillard.

```
                   P1       P2                  P2          P3         P4          P4    P5    P5 P6     P7       P8          P10  P11
Eco-btuB-RS    -----GCCGGUC-----CU-------GUG------AG-----UUAAU-AGGGAA--UCCAGUGCGAAUCUGGAGC-UG-ACGCGCAGCGGUAA-GGAAAGGUGCGAUGAUUGCGUUAUGCGGACACUGCCAU

Dre-Cbl-RS1    CGAGGGCAAGA--------------ACUGAAA----------------AGGGAAGU-CAG--UGAAUG-CUGACGCGGUACCCGCCACUGUACG-AG--------GGAGUCGAUCCACAAAAUGUCACUGG
Dre-Cbl-RS2    CAAAGCUCAGGU--CCAAA----GUCCGAGGAU--UUUGGU--UGAAAAGGGAA-GCAGG-UGCAAAU-CCUGCACGG-UCCCGCCGCUGUAAU-GG---GGAAAUGUUUCAUGAAUGCCACUGGUUUAUUC
Dre-Cbl-RS3    AGGUUGCAUAAU-----UU-------UGC------AA------UAAU-AGGGAA-ACAGG-UGAAAUG-CCUGUACGG-UCCCGCCGCCGUAA-GGU-----GGAGCAGAUUCAAAGUUGCCACUGUUUACA
Dre-Cbl-RS4    UUAAUCCCAGGUG-CCUCA-------GGA------UGAGG--AUAAU-AGGGAAGACCGG---GAAA--CCGGCGCGG-ACCCACCACUGUAUGCGG---AGACGAAUUCCGAAUGCCACUGGAUAUGAUC
Dre-Cbl-RS5    GCAGAACAGGUGUUGAAUGAAGCUGAUAAGCGUACCAUUCAAUGAAUAAGGAAAGUCGGG--UAGAAG-CCCGCGCGG-UCACGCCGCUGUGA-AAG-----UGGAGUUCUUCAGGUAUACCACUGGGAAA


                P11    P10P8          P12   P12     P7     P6     P3            P1                          P13          PK            P13
Eco-btuB-RS    UCGGUGGGAAGUCAUCAUCUCUUAGUAUCUUAGAUACCCCUCCAAGC-CCGAAGACCU----------GCCGGC--CAACGUCGCAUCU--GGUUCUCAUCA-------UCGCGUAAUAU---UGAUGAAACCUGC

Dre-Cbl-RS1    GAUUUGCCUGGGAAGACGUGAGUCGAUGAUGAU-------CUUGAGC-CAGGAAACCU----GC----UCUUGCC---AGCCGCUACGGCA-------ACCU-------ACGGGAUGUUAGGGAGGUGAAUACUCU
Dre-Cbl-RS2    CGGGAAGGCGUGAAACAUUGAUGAU---------------CCGGAGU-CAGAAGACCU----G-----CCUGAGUAGUUUACUUCAACUCUGCGAAAGACAG--------AGGAGGUG-----CUGUAUGUUAAAC
Dre-Cbl-RS3    AUGGGAAGGCUGAAUUUGCUUUGAA---------------ACCGAGU-CGGAAGACCUGCCUGAGCCAUUUUGC---------ACUGU---------UUUCA--------CGGAGGA------UGAAAGGUGUAGC
Dre-Cbl-RS4    UGGGAAGGCCGGGAGGAGAAUGAC----------------CCGUAAGCCAGGAGACCU----G-----CCUAGG----------AUUG--------GGAAGAUGAUGUAAUGGUAAGAACACUUCCUUCAAUGAUU
Dre-Cbl-RS5    CUGGGAAGGUCUGAAGAUCGAUGAUG-------------CUUAAGC-CAGAAGACCU----------ACCUGUU--UUAGUGUCUCACCAUAUA--CUCUA------CGAGUGA--------UAGAGAGGGGGAU


                   P14              P14         Met
Eco-btuB-RS    GGCAUCCUUCUUCUAUUGUGGAUGCUUUACAAUG

Dre-Cbl-RS1    GUGUUUUCUGGAUUCUUACCGUUUUUUGAUACAGGUUAGACUCUUUACCCUUUCAUGGUAAAGAGUUUUUUUGUUUUUCGGAUUUUGUUAUUAGCUAUGGAGGUUAAAAUG
Dre-Cbl-RS2    AUCAUAUAUUUUACUAAAUAUAUUGUUGACACCUCUUGACGUGAAGUUGAGAGGUGUUUUUGUUAAAGGAUAAAGAUGGAGGAAUUUUCAUG
Dre-Cbl-RS3    AUACGAAUCGUGCAGAAGUGUUUUAGACUGUUUGCAGAUUGUGUAAGUAAAACCUCUUCCAACCGCCGGUUAGAGAGGUUUUUUGAUGUUCAUGGAUGGACGAAUGCCGCGAUGCCAAGGAUGGCAAGGAGCGGCGAUG
Dre-Cbl-RS4    AAAUGACUUAGAAUGGGAAAGUCUCUUAGAAUAUAAGAGACUUUUGUUUUGUUUUAUUUAAAAAGAAUCGAAACAAAGGAGAAAUGAUUAUG
Dre-Cbl-RS5    GUUAUGUGCAAGACUACCGAGGAAAACUUCCUAGCAUAUGAGCCUCUCGACGUAAGAUGUUGAGAGGUUUUUUGUUUCAUAUCUUAUAUAGGAGAGUUUGACGAUG
```

Figure S8. Sequence analysis of *D. restrictus* cobalamin riboswitches. The five cobalamin riboswitches (Cbl-RS) were obtained using the Rfam toolbox, then automatically aligned using ClustalX together with *E. coli btuB* Cbl-RS, and finally manually refined to highlight the conserved secondary structures. The conserved hairpin structures are indicated by P1 to P14 colour shaded sequences. The terminal yellow shaded sequences in the lower panel indicate the putative transcriptional terminators located upstream of the ribosome-binding sequence (in blue) and the AUG start codon of the following gene (in bold).
