# Supplementary material for: Functional genomics of corrinoid starvation in the organohalide-respiring bacterium Dehalobacter restrictus strain PER-K23
Source: Front Microbiol. 2015 Jan 6;5:751. doi: 10.3389/fmicb.2014.00751 (PMC4285132; doi:10.3389/fmicb.2014.00751)
Supplement: Supplementary file 14 [file Image9.PDF]

Supplementary material

To the article 'Functional genomics of corrinoid starvation in the organohalide-respiring bacterium *Dehalobacter restrictus* strain PER-K23' by A. Rupakula, Y. Lu, T. Kruse, S. Boeren, C. Holliger, H. Smidt and J. Maillard.

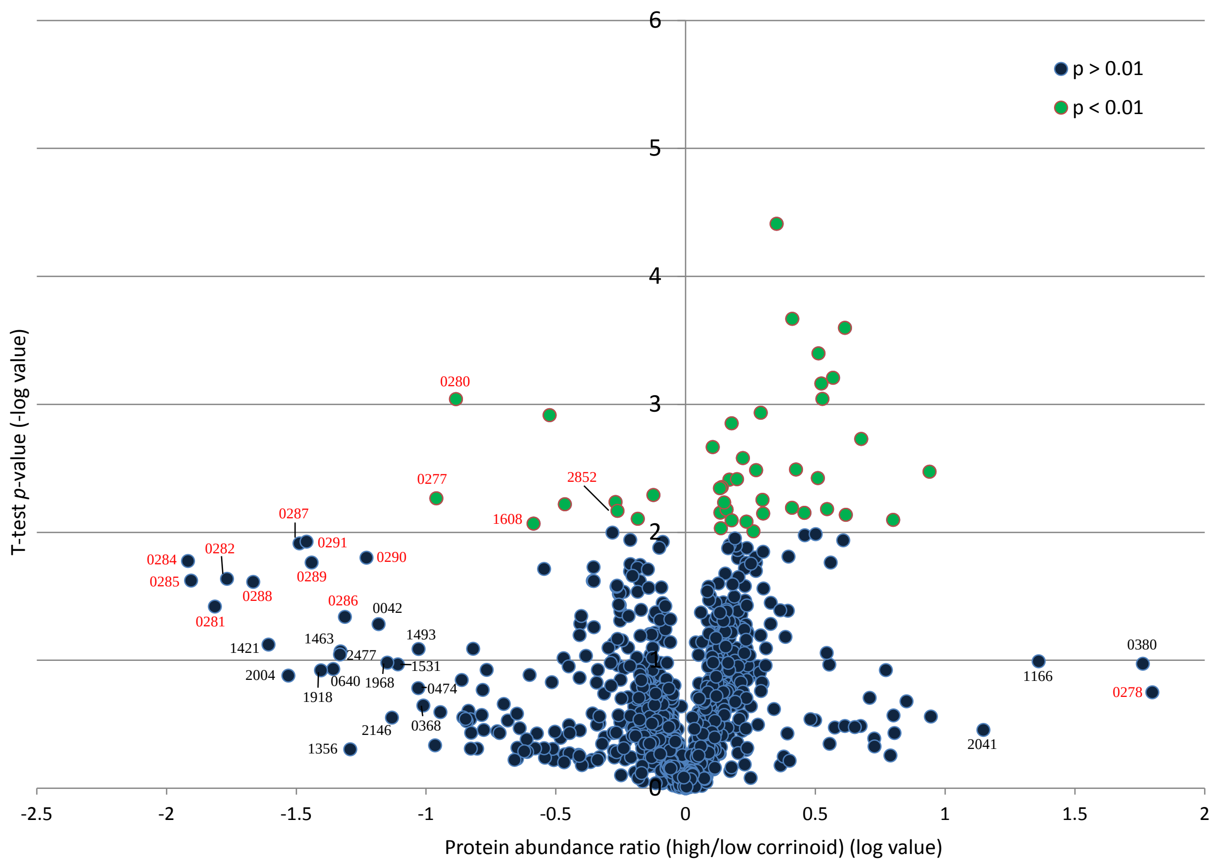

Figure S9. Volcano plot showing the whole-proteome data of *D. restrictus* cells cultivated with high (250 µg/L) *vs*. low (10 µg/L) cyanocobalamin in the medium. Differences between two cobalamin conditions with *p* value >0.01 are considered as non-significant by T-test (dark dots). Proteins with *p* value <0.01 are shown with green dots. Proteins associated with corrinoid metabolism showing a *p* value <0.01, or a *p* value >0.01 but a fold change higher than 10 are indicated with a red locus tag (the number corresponds to the Dehre_# locus tag used throughout the study), other proteins unrelated to corrinoid metabolism but with fold change higher than 10 are indicated with a black locus tag.
