# Supplementary material for: Functional genomics of corrinoid starvation in the organohalide-respiring bacterium Dehalobacter restrictus strain PER-K23
Source: Front Microbiol. 2015 Jan 6;5:751. doi: 10.3389/fmicb.2014.00751 (PMC4285132; doi:10.3389/fmicb.2014.00751)
Supplement: Supplementary file 8 [file Image3.PDF]

Supplementary material

To the article 'Functional genomics of corrinoid starvation in the organohalide-respiring bacterium *Dehalobacter restrictus* strain PER-K23' by A. Rupakula, Y. Lu, T. Kruse, S. Boeren, C. Holliger, H. Smidt and J. Maillard.

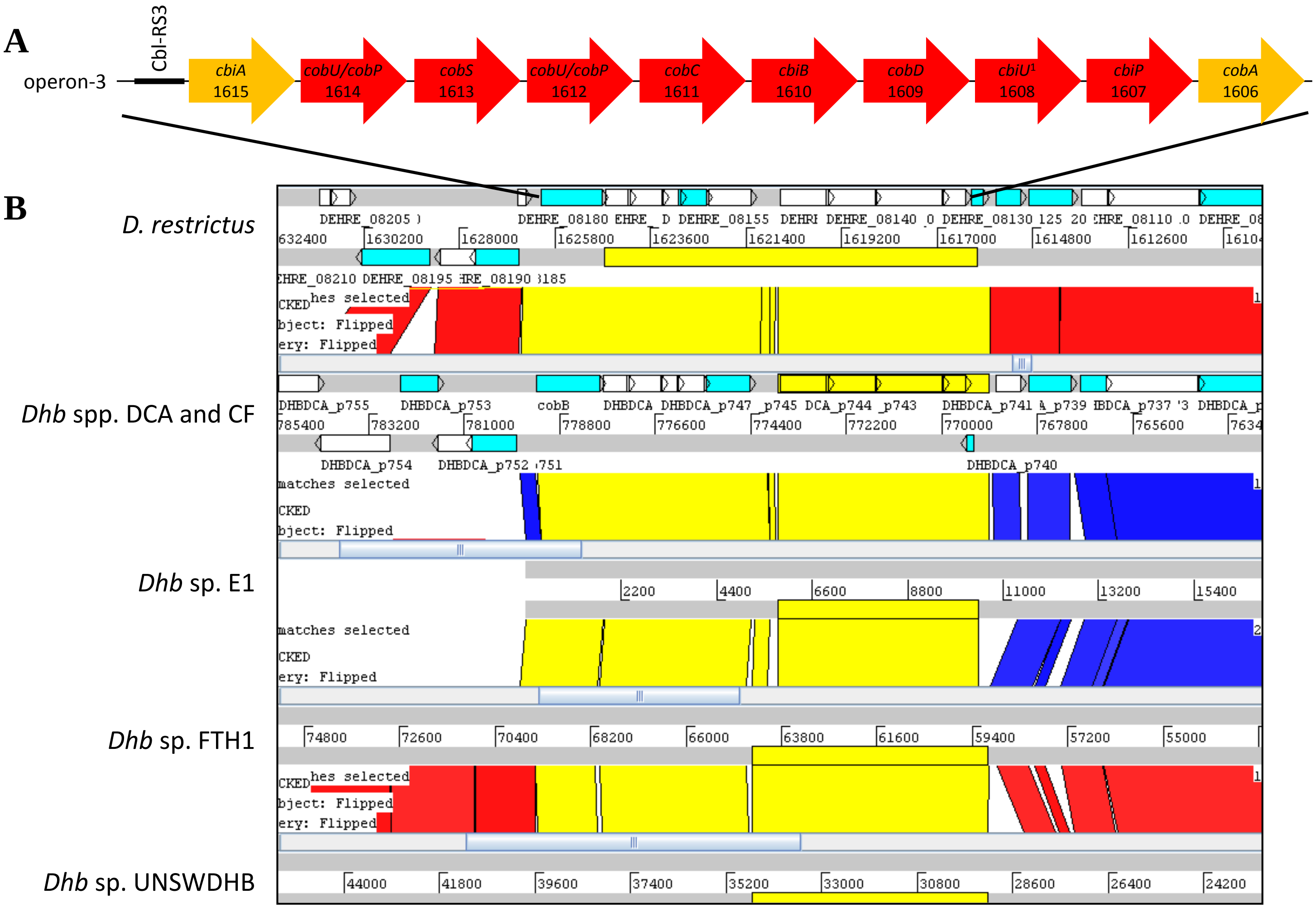

Figure S3. Synteny map of corrinoid operon-3 in *Dehalobacter* spp. (**A**) The gene array of operon-3 of *D. restrictus* is preceded by the cobalamin riboswitch Cbl-RS3. (**B**) The yellow boxes in the map indicate a nearly identical genetic structure in other studied *Dehalobacter* genomes.
